# racoon_clip—a complete pipeline for single-nucleotide analyses of iCLIP and eCLIP data

**DOI:** 10.1093/bioadv/vbae084

**Published:** 2024-06-26

**Authors:** Melina Klostermann, Kathi Zarnack

**Affiliations:** Faculty of Biosciences, Buchmann Institute for Molecular Life Sciences & Institute of Molecular Biosciences, Goethe University Frankfurt, Frankfurt am Main 60438, Germany; Faculty of Biosciences, Buchmann Institute for Molecular Life Sciences & Institute of Molecular Biosciences, Goethe University Frankfurt, Frankfurt am Main 60438, Germany

## Abstract

**Motivation:**

A vast variety of biological questions connected to RNA-binding proteins can be tackled with UV crosslinking and immunoprecipitation (CLIP) experiments. However, the processing and analysis of CLIP data are rather complex. Moreover, different types of CLIP experiments like iCLIP or eCLIP are often processed in different ways, reducing comparability between multiple experiments. Therefore, we aimed to build an easy-to-use computational tool for the processing of CLIP data that can be used for both iCLIP and eCLIP data, as well as data from other truncation-based CLIP methods.

**Results:**

Here, we introduce racoon_clip, a sustainable and fully automated pipeline for the complete processing of iCLIP and eCLIP data to extract RNA binding signal at single-nucleotide resolution. racoon_clip is easy to install and execute, with multiple pre-settings and fully customizable parameters, and outputs a conclusive summary report with visualizations and statistics for all analysis steps.

**Availability and implementation:**

racoon_clip is implemented as a Snakemake-powered command line tool (Snakemake version ≥7.22, Python version ≥3.9). The latest release can be downloaded from GitHub (https://github.com/ZarnackGroup/racoon_clip/tree/main) and installed via pip. A detailed documentation, including installation, usage, and customization, can be found at https://racoon-clip.readthedocs.io/en/latest/. The example datasets can be downloaded from the Short Read Archive (SRA; iCLIP: SRR5646576, SRR5646577, SRR5646578) or the ENCODE Project (eCLIP: ENCSR202BFN).

## 1 Introduction

**Figure 1.**
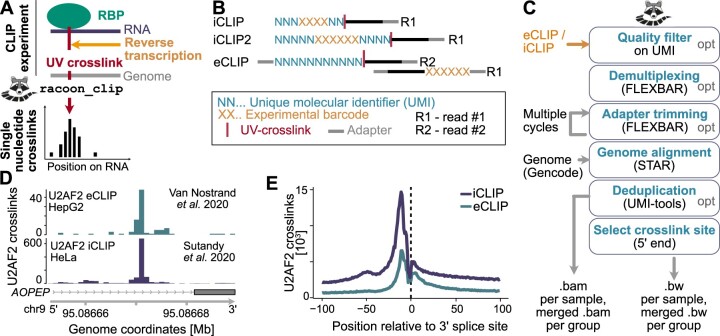
racoon_clip allows for efficient processing of iCLIP and eCLIP data. (A) Schematic of iCLIP/eCLIP experiments (left) to obtain single-nucleotide resolution (right). The RNA-binding protein (RPB, green) of interest is crosslinked to its *in vivo* RNA targets (dark blue) via UV irradiation. The RBP–RNA complex is immunoprecipitated with a specific antibody. Reverse transcription (orange arrow) of the RNA fragment will stop one nucleotide upstream of the UV crosslink site (red bar). By extracting this position, iCLIP/eCLIP data can be processed to single-nucleotide resolution. (B) Differences in the sequencing read composition between iCLIP, iCLIP2, and eCLIP experiments. All experiments carry the unique molecular identifier (UMI) at the 5′ end of the truncated cDNAs. However, since eCLIP usually employs paired-end sequencing, read #2 will contain the truncation site. The experimental barcode in iCLIP and iCLIP2 is positioned between the UMIs, while it is separately positioned in read #1 in eCLIP. (C) Steps performed by racoon_clip. (D) Exemplary crosslink profiles of U2AF2 eCLIP (Van Nostrand *et al.* 2020) (top) and U2AF2 iCLIP (Sutandy *et al.* 2018) (bottom) after racoon_clip processing. (E) Metaprofile of U2AF2 crosslink events from eCLIP (petrol) and iCLIP (dark blue) data around annotated 3′ splice sites.

UV crosslinking and immunoprecipitation (CLIP) coupled to high-throughput sequencing has become a popular tool to query the RNA-binding behavior of RNA-binding proteins (RBPs) in a transcriptome-wide manner (Lee and Ule 2018). CLIP protocols use UV irradiation to crosslink the RBPs to their bound RNAs *in vivo* and then extract the RBP–RNA complexes via immunoprecipitation with a specific antibody. Two common variants of CLIP, named individual-nucleotide resolution CLIP (iCLIP) (König *et al.* 2010) and enhanced CLIP (eCLIP) (Van Nostrand *et al.* 2016), detect the crosslink sites of RBPs on the RNAs by capturing the truncation of reverse transcription at these sites (Fig. 1A). From these data, the crosslink position of the RBP can be extracted with single-nucleotide resolution as the crosslink sites reside one nucleotide (nt) upstream of the 5′ ends of the reads (Haberman *et al.* 2017). Still, there are differences between the protocols like the positioning and design of experimental barcodes and unique molecular identifiers (UMIs) or the preferred usage of single-end versus paired-end sequencing (Fig. 1B, Table 1). Therefore, while the results of iCLIP and eCLIP experiments should be very comparable from an experimental point of view, differences in the experimental design and the resulting read architecture must be considered in the computational analysis (Buchbender *et al.* 2020).

**Table 1.** Differences of iCLIP and eCLIP data types that are relevant for data processing.

	iCLIP (König *et al.* 2010)	iCLIP2 (Buchbender *et al.* 2020)	eCLIP (Van Nostrand *et al.* 2016)	eCLIP downloaded from ENCODE
Library strategy	Single-end reads	Single-end reads	Paired-end reads (read #2 contains crosslink)	Paired-end reads (read #2 contains crosslink)
UMI (N) and barcode (X) setup	N_3_X_4_N_2_	N_5_X_6_N_4_	Read #2: N_5_ or N_10_	Read #2: N_5_ or N_10_ already removed from sequence and stored in read name

Here, we present the command line tool racoon_clip that implements single-nucleotide resolution processing for iCLIP and eCLIP data. It is furthermore adaptable to related technologies dependent on reverse transcription truncations that use different adapter configurations via the customization option. We note that racoon_clip cannot process data from technologies that depend on read-through across the crosslink site such as CRAC, PAR-CLIP, or HITS-CLIP (Lee and Ule 2018). The automated pipeline is based on the previously published workflow (Busch *et al.* 2020) that was extended to cater both iCLIP and eCLIP data. Being built from a Snakemake pipeline (Mölder *et al.* 2021), racoon_clip can be run in a fully automated and multi-threaded manner. Furthermore, it can be easily installed via download from GitHub and local installation via pip. Together, this makes racoon_clip an easy-to-use option to obtain single-nucleotide information on RBP crosslink sites from iCLIP and eCLIP data.

## 2 Methods

racoon_clip processes raw iCLIP or eCLIP sequencing reads to obtain single-nucleotide RBP crosslink sites. It is a command line tool powered by a Snakemake pipeline implemented in Python, bash, and R and uses publicly available command line tools where possible. racoon_clip can be downloaded from GitHub (https://racoon-clip.readthedocs.io/en/latest/) and installed via pip, including all dependencies.

racoon_clip can be run on data from different CLIP protocols by either specifying one of the pre-set experiment types (“iCLIP,” “iCLIP2,” “eCLIP_5ntUMI,” “eCLIP_10ntUMI,” “eCLIP_ENCODE_5ntUMI,” “eCLIP_ENCODE_10ntUMI,” or “noBarcode_noUMI”) with the *experiment_type* parameter or using a custom barcode and UMI setup. As input, racoon_clip takes the sequencing reads as multiplexed or demultiplexed FASTQ files, the genome assembly as FASTA file, and the gene annotation as GTF file. All steps performed by racoon_clip are fully customizable and can be specified either via a configuration (config) file or directly in the command line (see User manual). The output of racoon_clip includes the aligned reads in BAM format, the single-nucleotide crosslink events in BED and BIGWIG format, as well as a detailed processing report in HTML format.

### 2.1 Performed steps

The racoon_clip pipeline consists of three major steps (Fig. 1C): (i) the preprocessing of the sequencing reads, (ii) the genomic alignment, and (iii) the extraction of crosslink events. In the preprocessing step, racoon_clip deals with the barcodes of the iCLIP or eCLIP experiment, taking into account multiple barcode formats. In brief, the FASTQ files are demultiplexed, the barcodes are trimmed off, and the UMIs are stored in the read names. To perform this on data from different protocols, racoon_clip offers predesigned settings for iCLIP, iCLIP2, eCLIP, or eCLIP downloaded from ENCODE and a flexible architecture of experimental barcodes with the basic structure U_UMI1_len_–B_barcode_len_–V_UMI2_len_ for custom read designs.

racoon_clip can perform barcode and/or adapter trimming and/or demultiplexing using FLEXBAR (version 3.5.0) (references for this and all further tools employed by racoon_clip can be found in Busch *et al.* 2020). These options can be specified depending on the input data. During barcode trimming, the UMIs are appended to the read names for later deduplication. If present, barcodes need to be provided in a FASTA file. To ensure correct barcode and UMI assignment, racoon_clip can perform an optional quality filtering on the barcode regions (*quality_filter_barcodes*) using awk (mawk version 1.3.3). Of note, there is a specialized option for eCLIP data directly downloaded from the ENCODE website, as ENCODE provides preprocessed sequencing reads without barcodes and with UMIs already stored in the read name, albeit in a different position. For adapter trimming, custom adapters can be provided as a FASTA file. Otherwise, a standard set of sequencing adapters from Illumina and eCLIP adapters is used. By default, one round of adapter trimming is performed but multiple cycles can be chosen. For example, two cycles are recommended for ENCODE eCLIP data (https://github.com/yeolab/eclip). We advise to check in the HTML report that adapters have been trimmed off correctly. The output of the preprocessing step is demultiplexed FASTQ files, containing the sequencing reads of the individual samples without adapters and barcodes and with the UMIs appended to the read names.

In the second step, the demultiplexed reads are aligned to the genome and then deduplicated. For genomic alignment using STAR (version 2.7.10), the FASTA file of the genome assembly and the corresponding GTF file of the gene annotation need to be provided. Soft-clipping is restricted to the 3′ end of the reads (--alignEndsType “Extend5pOfRead1”) to preserve the exact crosslink position upstream of the 5′ end. All other STAR parameters can be customized, for instance to allow for multi-mapping reads. By default, STAR is configured to output only unique alignments (--outFilterMultimapNmax 1) with up to 4% mismatches (--outFilterMismatchNoverReadLmax 0.04). Next, the aligned reads are deduplicated by removing reads mapping at the same location and sharing the same UMI using UMI-tools (version 1.1.1). There is an option to skip deduplication (*deduplicate: False*), although we recommend performing deduplication if possible. The option to skip deduplication can be used for very deep data when UMIs start going into saturation as observed for example for *in vitro* iCLIP (Sutandy *et al.* 2018). Furthermore, deduplication needs to be skipped when UMI information is not available as for example in some SRA-stored raw datasets where data is stored without the original read names resulting in loss of UMI information. Finally, the BAM files are indexed using SAMtools (version 1.11). Ultimately, the second step returns aligned and deduplicated reads in BAM format.

In the third step of racoon_clip, the crosslink sites are extracted by selecting the position 1 nt upstream of the 5′ end of the aligned reads using BEDTools (version 2.30.0) and kentUtils (version 377). The crosslink events are output in BED and BIGWIG format.

Once completed, racoon_clip provides an HTML processing report that summarizes the data quality (using FastQC (version 0.12) and MultiQC (version 1.14)) and relevant statistics for each step. This allows the user to assess the quality of the processed dataset and ensure that all steps were performed as expected.

## 3 Results

To showcase the functionalities of racoon_clip, we processed an eCLIP dataset from ENCODE (Van Nostrand *et al.* 2020) (2 replicates, 18 760 220 reads in total; ENCODE ID: ENCSR202BFN) and an iCLIP dataset from a previous publication (Sutandy *et al.* 2018) (3 replicates, 66 041 912 reads in total; SRA ID: SRR5646576, SRR5646577, SRR5646578) for the splicing factor U2AF2. For the iCLIP dataset, racoon_clip needed 1 h and 54 min at a peak RAM usage of 40.5 Gigabytes on 6 cores. As the data is stored at SRA already demultiplexed, deduplicated, and with adapters and barcodes trimmed, we used the following settings for the processing: *experiment_type: iCLIP, demultiplex: False, quality_filter_barcodes: False, adapter_trimming: False, deduplicate: False*.

For the eCLIP dataset, 2 h and 13 min at a peak RAM usage of 35.4 Gigabytes were needed on 6 cores. The following settings were used: *experiment_type: eCLIP_ENCODE_10ntUMI, demultiplex: False, quality_filter_barcodes: False, adapter_trimming: True, adapter_cycles: 2, deduplicate: True*.

From the iCLIP data, 95.8% of the sequencing reads were uniquely aligned. In total, racoon_clip detected 63 266 600 crosslink events for the merged replicates. Similarly, for the eCLIP data, 67.2% were uniquely mapped, and of these 81.2% were kept after deduplication, yielding a total of 10 282 489 crosslink events.

Exemplary U2AF2 crosslink profiles from the iCLIP and eCLIP data on the *AOPEP* transcript are shown in Fig. 1D. The unified processing by racoon_clip makes both datasets directly comparable. As expected, the U2AF2 crosslink events accumulate upstream of 3′ splice sites (Fig. 1E), underlining the high resolution of the RBP crosslink profiles.

## 4 Conclusion

In summary, racoon_clip enables users to obtain single-nucleotide resolution signal from their eCLIP and iCLIP or comparable data. The output files can directly be used as input for binding site definition as described in Busch *et al.* 2020, using for example the peak calling software PureCLIP (Krakau *et al.* 2017) and R/Bioconductor package BindingSiteFinder (Brüggemann and Zarnack 2021). racoon_clip thereby builds a solid foundation for in-depth eCLIP and iCLIP data analyses and facilitates comparisons of multiple experiments from rich resources such as the ENCODE eCLIP data (Van Nostrand *et al.* 2020).

## Data Availability

There are no new data associated with this article.
